# Unhealthy behaviors and risk of uncontrolled hypertension among treated individuals-The CONSTANCES population-based study

**DOI:** 10.1038/s41598-020-58685-1

**Published:** 2020-02-05

**Authors:** Michelle Cherfan, Alexandre Vallée, Sofiane Kab, Pascale Salameh, Marcel Goldberg, Marie Zins, Jacques Blacher

**Affiliations:** 1 Nutritional Epidemiology Research Unit (EREN), Inserm U1153, Inra U1125, Cnam, Crnh, Paris 13 University Sorbonne Paris Cite, Bobigny, France; 2 Faculty of Pharmacy, Lebanese International University, Beirut, Lebanon; 3 Faculty of Medicine, Paris-Descartes University, Paris, France; 4 Diagnosis and Therapeutic Center, Hypertension and Cardiovascular Prevention Unit, Hôtel-Dieu Hospital; AP-HP, Paris, France; 5 Population-based Epidemiological Cohorts Unit, Inserm, UMS011 Villejuif, France; 6 Faculty of Public Health, Lebanese University, Fanar, Lebanon; 7 Institut National de Santé Publique, Epidémiologie Clinique et Toxicologie (INSPECT-LB), Beirut, Lebanon

**Keywords:** Hypertension, Risk factors

## Abstract

From an epidemiological standpoint, quantifying the individual and the combined effect of lifestyle factors on uncontrolled blood pressure (BP) deserves further evaluation. We aimed to examine the individual and combined associations between unhealthy behaviors and uncontrolled hypertension among treated hypertensive adults. Cross-sectional analysis was conducted using data from CONSTANCES, an ongoing French population-based cohort study. Uncontrolled BP was defined as mean systolic BP ≥140 mmHg and/or mean diastolic BP ≥90 mmHg. Unhealthy behaviors were considered as heavy alcohol consumption, low or medium adherence to dietary recommendations, sedentary physical activity level, and overweight. A total of 10,710 hypertensive treated volunteer participants were included and 56.1% had uncontrolled hypertension; of them, 2.0%, 24.5%, 54.0% and 19.5% exhibited 0, 1, 2 or ≥3 unhealthy behaviors respectively. In men, there was an increased odds of uncontrolled hypertension with heavy alcohol drinking compared to light-or-never (adjusted odds ratio 1.34, 95% CI 1.10–1.63), with low as well as with medium adherence to dietary recommendations compared to high (p < 0.05 for both), and with overweight or obesity compared to a normal body mass index (p ≤ 0.001 for both). In addition, men reporting a combination of ≥3 unhealthy behaviors compared to none, had an increased odds of hypertension of 1.67 (95% CI 1.09–2.53). Unhealthy behaviors described as, heavy alcohol consumption, non-adherence to dietary recommendations and overweight are associated with uncontrolled hypertension, at the individual and combined level, and particularly in men. Improvement of modifiable lifestyle factors could offer considerable benefits in the management of hypertension.

## Introduction

Arterial hypertension is a global public health issue affecting more than 1 billion individuals worldwide and causing an estimated ten million deaths every year^1^. Despite the availability of efficient and well-tolerated medications and widespread public health efforts to treat individuals with hypertension, inadequate blood pressure (BP) control is frequently reported among treated hypertensive individuals, and contributes significantly to increased risk of cardiovascular disease (CVD), stroke and chronic kidney disease (CKD)^2^.

A number of epidemiological studies commonly reported a high prevalence rate of uncontrolled hypertension at the 140/90 mmHg threshold; in the United States, data analysis from the National Health and Nutrition Examination Survey (NHANES) found that among treated individuals around 45% had uncontrolled BP^3^. Within Europe, BP control rate among those treated reached 40% in England, 30% in Germany, 28% in Italy, 19% in Spain and 21% in Sweden^4^. Similarly, the estimated prevalence of hypertension in France is 31% and 51.3% of hypertensive treated patients are not controlled^5^.

A broad range of factors have been identified that contribute to poor BP control. These include, physician inertia (i.e. lack of therapeutic action when the patient’s BP is uncontrolled)^6^, deficiencies of healthcare systems in their global approach to chronic diseases^7^ and low adherence to treatment including antihypertensive prescriptions and lifestyle changes^8^. In addition, factors such as socio-economic characteristics and poor lifestyle behaviors have been described as predictors of poor BP control^9^. Studies suggest that unhealthy lifestyle behaviors including heavy alcohol drinking, lack of physical activity, poor dietary habits and overweight may contribute to inadequate BP control among hypertensive treated individuals^9,10^. Alternatively, lifestyle changes were associated with decreased BP among hypertensive individuals;^11,12^ Appel *et al*., reported that behavioral interventions comprising increased physical activity, limitation of dietary sodium intake, reduced alcohol consumption, and weight loss, reduced systolic BP by 12.5 mmHg and diastolic BP by 5.8 mmHg^12^.

Several lifestyle modifications or non-pharmacological approaches are widely recommended in worldwide guidelines for the management and prevention of hypertension. These interventions target dietary habits, salt intake, potassium intake, alcohol consumption, physical activity and weight. However the quantitative or qualitative targets for each of these measures differ across the guidelines^13,14^. This heterogeneity makes their promotion more challenging and justifies the need to conduct further studies evaluating their impact on different populations. In fact, these recommendations emphasize lifestyle changes based on intervention trials that were especially effective in pre- or hypertensive individuals; hence, the study of their effect in terms of improving BP control in hypertensive treated individuals remains necessary.

As such, from an epidemiological perspective, examining the quantitative effect of unhealthy lifestyle factors on uncontrolled hypertension, at the individual and combined level, warrants further evaluation. The French nationwide large population-based study, CONSTANCES^15^, which is designed to contribute to the development of epidemiologic research, serve as an opportunity to provide additional data on this subject. Hence, this ancillary study was conducted to evaluate the individual and collective relationship between unhealthy behaviors, particularly, heavy alcohol consumption, low physical activity, non-adherence to dietary recommendations, and overweight, with uncontrolled hypertension. We aimed to assess the quantitative extent to which modifiable lifestyle factors are determinants of uncontrolled hypertension in order to assess the magnitude of their effect in the management of hypertension, from a gender-based perspective.

## Results

### Baseline characteristics of study population

Table 1 presents the baseline characteristics of the studied participants, which were compared among subjects with controlled and uncontrolled hypertension. The mean ± standard deviation (SD) age of the population was 59.8 ± 8.6. Unhealthy behaviors were prominent; the majority of the participants (91.3%) did not highly adhere to dietary recommendations, more than two third (70%) were overweight (body mass index ≥25), 15% consumed alcohol heavily and 10.2% were sedentary.

**Table 1:** Frequency of uncontrolled hypertension according to characteristics of participants.

Characteristic	All participants n, (%)	Uncontrolled hypertension n, (%)	Controlled hypertension n, (%)	P value
**Overall**	**10710 (100)**	**6003 (56.1)**	**4707 (43.9)**	
**Gender**				
*Male*	6032 (56.3)	3776 (62.9)	2256 (47.9)	<0.001
*Female*	4678 (43.7)	2227 (37.1)	2451 (52.1)	
Age, year, mean	59.8 ± 8.6	61.0 ± 7.7	58.2 ± 9.3	<0.001
**Age, year**				<0.001
*[18–39]*	342 (3.2)	107 (1.8)	235 (5.0)	
*[40–49]*	1064 (9.9)	471 (7.8)	593 (12.6)	
*[50–59]*	3053 (28.5)	1596 (26.6)	1457 (30.9)	
≥*60*	6251 (58.4)	3829 (63.8)	2422 (51.5)	
Systolic BP, mmHg	142.6 ± 17.4	154.4 ± 12.7	127.5 ± 8.7	<0.001
Diastolic BP, mmHg	81.8 ± 9.9	86.7 ± 9.0	75.6 ± 7.2	<0.001
Heart rate, beats per min	65 ± 11	66 ± 12	65 ± 11	<0.001
Serum creatinine (mmol/l)	78.3 ± 21.5	78.9 ± 19.9	77.4 ± 23.3	0.001
**Education level**				<0.001
≤*high school diploma*	4754 (44.4)	2812 (46.8)	1942 (41.3)	
*Undergraduate degree*	1693 (15.8)	947 (15.8)	746 (15.8)	
*Postgraduate degree*	4263 (39.8)	2244 (37.4)	2019 (42.9)	
**Income of the house/month**				0.615
*Less than 1000 €*	504 (4.7)	269 (4.5)	235 (5.0)	
*1000–2099 €*	2287 (21.3)	1279 (21.3)	1008 (21.4)	
*2100–4199 €*	5201 (48.6)	2935 (48.9)	2266 (48.1)	
*More or equal than 4200 €*	2718 (25.4)	1520 (25.3)	1198 (25.5)	
**Familial situation**				<0.001
*Single*	2554 (23.8)	1319 (22.0)	1235 (26.2)	
*Couple life*	8156 (76.2)	4684 (78.0)	3472 (73.8)	
Oral contraceptive or HRT*	958 (20.5)*	434 (19.5)*	524 (21.4)*	0.058
BMI (Kg/m^**2**^)	28.1 ± 5.0	28.3 ± 4.9	27.7 ± 5.0	<0.001
**BMI class**				<0.001
*<25*	3101 (29.0)	1568 (26.1)	1533 (32.6)	
*25.0*–*29.9*	4364 (40.7)	2529 (42.1)	1835 (39.0)	
≥*30.0*	3245 (30.3)	1906 (31.8)	1339 (28.4)	
**Physical activity**				0.008
*Sedentary*	1095 (10.2)	586 (9.8)	509 (10.8)	
*Moderate*	3928 (36.7)	2152 (35.8)	1776 (37.7)	
*High*	5687 (53.1)	3265 (54.4)	2422 (51.5)	
DASH score	26.1 ± 3.7	26.0 ± 3.7	26.3 ± 3.7	<0.001
**DASH categories**				<0.001
*Low*	1361 (12.7)	750 (12.5)	611 (13.0)	
*Medium*	8413 (78.6)	4766 (79.4)	3647 (77.5)	
*High*	936 (8.7)	487 (8.1)	449 (9.5)	
**Alcohol consumption**				<0.001
*Never/light*	1828 (17.1)	931 (15.5)	897 (19.1)	
*Moderate*	7271 (67.9)	4053 (67.5)	3218 (68.4)	
*Heavy*	1611 (15.0)	1019 (17.0)	592 (12.6)	
**Smoking status**				<0.001
*Non-smoker*	4987 (46.6)	2740 (45.6)	2247 (47.7)	
*Current smoker*	1200 (11.2)	584 (9.7)	616 (13.1)	
*Ex-smoker*	4523 (42.2)	2679 (44.7)	1844 (39.2)	
History of CV events	1401 (13.1)	709 (11.8)	692 (14.7)	<0.001
Diabetes	1661 (15.5)	1045 (17.4)	616 (13.1)	<0.001
Dyslipidemia	6418 (59.9)	3733 (62.2)	2685 (57.0)	<0.001
Chronic kidney disease	176 (1.7)	99 (1.6)	78 (1.7)	0.345
**Anti-hypertensive medications**				0.001
*Mono-therapy*	5932 (56.3)	3227 (54.6)	2705 (58.4)	
*Dual therapy*	3619 (34.3)	2081 (35.2)	1538 (33.2)	
*Triple therapy or more*	995 (9.4)	603 (10.2)	392 (8.4)	

Data are mean ±SD for quantitative variables or percent for categorical.

*P* from logistic regression model adjusted for age and sex.

*Frequency among women only.

Abbreviations: BMI, body mass index (Kg/m^2^); BP, blood pressure; CV, cardiovascular; DASH, dietary approach to stop hypertension; HRT, hormone replacement therapy; SD, standard deviation.

Among the 10710 hypertensive treated participants 6003 had uncontrolled hypertension, reaching a prevalence of poor BP control of 56.1%. Uncontrolled hypertension was more prevalent in men than in women (62.9% vs. 47.9% respectively, p < 0.001) and with increased age categories with the highest prevalence seen in those more than 65 years old (63.1%). After adjustment to age and gender, uncontrolled hypertension was more frequent in participants with lower education (46.8% vs. 41.3%, p < 0.001), living in couple (78% vs. 73.8%, p < 0.001), with diabetes (17.4% vs. 13.1%, p < 0.001) or with dyslipidemia (62.2% vs. 57%, p < 0.001). It was less common in those with history of CVD (11.8% vs. 14.7, p < 0.001). As for lifestyle factors, those who are overweight or obesity and those with heavy alcohol consumption have a significantly higher prevalence of uncontrolled hypertension (p < 0.001 for both variables) while high dietary adherence is associated with lower frequency. Interestingly, inverse associations were seen with physical activity and smoking status, those with high-level physical activity (p = 0.008) had more often uncontrolled hypertension, whereas current smokers seem to have more often controlled hypertension (p < 0.001). Globally, 56.3% of the study subjects were receiving one anti-hypertensive medication, while 34.3% were on dual therapy and 9.4% were using three medications or more; those with uncontrolled hypertension were less likely to be receiving mono-therapy and more likely to be receiving dual or triple (or more) therapy (p = 0.001).

### Characteristics and unhealthy behaviors

**Figure 1:**
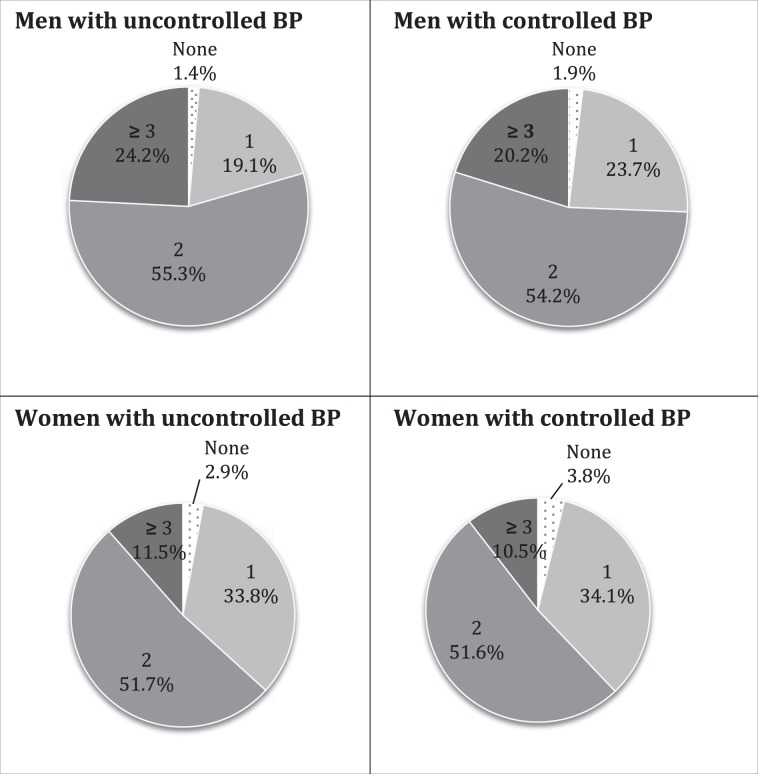
Proportion of subjects by number of unhealthy behaviors stratified by sex.

Figure 1 illustrates the frequency of unhealthy behavior(s) in participants with and without uncontrolled hypertension. Also, Tables 2 and 3 present, respectively, men and women’s characteristics according to the number of unhealthy behaviors. Globally, unhealthy behaviors were more commonly seen in men, whereby 22.7% reported having three or more unhealthy behaviors compared to 11% of women (p < 0.001). Among participants with uncontrolled hypertension, 2.0%, 24.5%, 54.0% and 19.5% exhibited 0, 1, 2 or ≥3 unhealthy behaviors, respectively. Between sexes, minimal differences were seen in the studied associations; age-adjusted analysis found an increasing number of unhealthy behaviors associated with a lower household monthly income (p < 0.001), lower education (p < 0.001), presence of dyslipidemia or diabetes (p trend < 0.001), and current smoking status (p < 0.001).

**Table 2:** Men’s characteristics according to the number of unhealthy behaviors.

Number of unhealthy behaviors	0	1	2	3 or more	P value
Overall, n (%)	**97 (1.6)**	**1255 (20.8)**	**3312 (54.9)**	**1368 (22.7)**	—
Age, year, mean	60.9 ± 8.6	60.1 ± 8.9	60.2 ± 8.1	60.1 ± 7.9	0.767
**Age, year**					0.003
*[18–39]*	3 (3.1)	52 (4.1)	78 (2.4)	24 (1.8)	
*[40–49]*	7 (7.2)	106 (8.5)	312 (9.4)	134 (9.8)	
*[50–59]*	22 (22.7)	329 (26.2)	947 (28.6)	420 (30.7)	
≥*60*	65 (67.0)	768 (61.2)	1975 (59.6)	790 (57.7)	
Systolic BP	144.6 ± 17.8	143.5 ± 17.3	145.3 ± 16.5	146.0 ± 16.2	0.001
Diastolic BP	81.9 ± 10.0	81.8 ± 9.6	83.6 ± 9.8	84.2 ± 9.8	<0.001
Uncontrolled BP	53 (54.6)	721 (57.5)	2089 (63.1)	913 (66.7)	<0.001
Heart rate, beats per min	59 ± 9	62 ± 11	65 ± 11	67 ± 12	<0.001
Serum creatinine (mmol/l)	87.4 ± 35.3	85.3 ± 20.9	86.6 ± 22.3	85.3 ± 23.4	0.184
**Education level**					<0.001
≤*high school diploma*	33 (34.0)	462 (36.8)	1573 (47.5)	661 (48.3)	
*Undergraduate degree*	11 (11.3)	192 (15.3)	472 (14.3)	190 (13.9)	
*Postgraduate degree*	53 (54.6)	601 (47.9)	1267 (38.2)	517 (37.8)	
**Income of the house/month**					<0.001
*Less than 1000 €*	6 (6.2)	48 (3.8)	116 (3.5)	89 (6.5)	
*1000–2099 €*	16 (16.5)	195 (15.5)	635 (19.2)	304 (22.2)	
*2100–4199 €*	41 (42.3)	564 (45.0)	1670 (50.4)	597 (43.7)	
*More or equal than 4200 €*	34 (35.0)	448 (35.7)	891 (26.9)	378 (27.6)	
**Familial situation**					0.001
*Single*	22 (22.7)	228 (18.2)	595 (18.0)	314 (22.9)	
*Couple life*	75 (77.3)	1027 (81.8)	2717 (82.0)	1054 (77.1)	
BMI (Kg/m^**2**^)	23.2 ± 1.4	24.7 ± 3.4	29.2 ± 3.9	30.1 ± 4.0	<0.001
**BMI class**					<0.001
<25	97 (100)	965 (76.9)	250 (7.5)	27 (2.0)	
*25.0*–*29.9*	0 (0)	198 (15.8)	1904 (57.5)	752 (55.0)	
≥*30.0*	0 (0)	92 (7.3)	1158 (35.0)	589 (43.0)	
**Physical activity**					<0.001
*Sedentary*	0 (0)	2 (0.2)	115 (3.5)	553 (40.4)	
*Moderate*	28 (28.9)	450 (35.9)	1407 (42.5)	352 (25.7)	
*High*	69 (71.1)	803 (64.0)	1790 (54.0)	463 (33.9)	
DASH score	32.1 ± 1.8	27.1 ± 3.7	25.1 ± 3.3	23.8 ± 3.4	<0.001
**DASH categories**					<0.001
*Low*	0 (0)	72 (5.7)	408 (12.3)	284 (20.8)	
*Medium*	0 (0)	879 (70.0)	2829 (85.4)	1079 (78.9)	
*High*	97 (100)	304 (24.2)	75 (2.3)	5 (0.4)	
**Alcohol consumption**					<0.001
*Never/light*	20 (30.6)	174 (13.9)	443 (13.4)	89 (6.5)	
*Moderate*	77 (79.4)	1069 (85.2)	2659 (80.3)	322 (23.5)	
*Heavy*	0 (0)	12 (0.9)	210 (6.3)	957 (70.0)	
**Smoking status**					<0.001
*Non-smoker*	45 (46.4)	549 (43.7)	1229 (37.1)	389 (28.4)	
*Current smoker*	13 (13.4)	110 (8.8)	350 (10.6)	225 (16.5)	
*Ex-smoker*	39 (40.2)	596 (47.5)	1733 (52.3)	754 (55.1)	
History of CV events	20 (20.6)	235 (18.7)	585 (17.7)	259 (18.9)	0.630
Diabetes	9 (9.3)	150 (11.9)	687 (20.7)	322 (23.5)	<0.001
Dyslipidemia	55 (56.7)	686 (54.7)	2225 (67.2)	993 (72.6)	<0.001
Chronic kidney disease	5 (5.2)	21 (1.7)	64 (1.9)	23 (1.7)	0.191
**Anti-hypertensive medications**					<0.001
*Mono-therapy*	59 (62.1)	746 (60.2)	1660 (50.8)	638 (47.2)	
*Dual therapy*	25 (26.3)	408 (32.9)	1247 (38.2)	531 (39.3)	
*Triple therapy or more*	11 (11.6)	85 (6.9)	362 (11.0)	183 (13.5)	

Data are mean ± SD for quantitative variables or percent for categorical.

*P* from logistic regression model adjusted for age.

Abbreviations: BMI, body mass index (Kg/m^2^); BP, blood pressure; CV, cardiovascular; DASH, dietary approach to stop hypertension; SD, standard deviation.

**Table 3:** Women’s characteristics according to the number of unhealthy behaviors.

Number of unhealthy behaviors	0	1	2	3 or more	P value
Overall, n (%)	**157 (3.4)**	**1590 (34.0)**	**2416 (51.6)**	**515 (11.0)**	—
Age, year, mean	60.2 ± 9.2	59.2 ± 9.2	59.3 ± 8.8	59.4 ± 8.8	0.595
**Age, year**					0.476
*[18–39]*	6 (3.8)	72 (4.5)	92 (3.8)	15 (2.9)	
*[40–49]*	11 (7.0)	170 (10.7)	265 (11.0)	59 (11.5)	
*[50–59]*	50 (31.9)	429 (27.0)	705 (29.2)	151 (29.3)	
≥*60*	90 (57.3)	919 (57.8)	1354 (56.0)	290 (56.3)	
Systolic BP	138.1 ± 17.9	138.8 ± 18.6	139.6 ± 17.4	140.2 ± 16.9	0.271
Diastolic BP	77.0 ± 9.0	78.9 ± 10.1	80.4 ± 9.6	81.3 ± 9.5	<0.001
Uncontrolled BP	65 (41.4)	753 (47.4)	1152 (47.7)	257 (49.9)	0.312
Heart rate, beats per min	64 ± 9	65 ± 10	67 ± 11	68 ± 11	<0.001
Serum creatinine (mmol/l)	68.1 ± 14.4	68.0 ± 13.2	68.9 ± 10.5	68.3 ± 10.9	0.675
**Education level**					<0.001
≤*high school diploma*	44 (28.0)	581 (36.5)	1162 (48.1)	238 (46.2)	
*Undergraduate degree*	32 (20.4)	292 (18.4)	422 (17.5)	82 (15.9)	
*Postgraduate degree*	81 (51.6)	717 (45.1)	832 (34.4)	195 (37.9)	
**Income of the house/month**					<0.001
*Less than 1000 €*	7 (4.5)	67 (4.2)	125 (5.2)	46 (8.9)	
*1000–2099 €*	26 (16.5)	332 (20.9)	656 (27.2)	123 (23.9)	
*2100–4199 €*	75 (47.8)	804 (50.6)	1209 (50.0)	241 (46.8)	
*More or equal than 4200 €*	49 (31.2)	387 (24.3)	426 (17.6)	105 (20.4)	
**Familial situation**					<0.001
*Single*	69 (44.0)	449 (28.2)	709 (29.4)	168 (32.6)	
*Couple life*	88 (56.0)	1141 (71.8)	1707 (70.6)	347 (67.4)	
Oral contraceptive or HRT	36 (22.9)	392 (24.4)	449 (18.4)	81 (14.4)	<0.001
BMI (Kg/m^**2**^)	22.2 ± 1.7	23.7 ± 3.6	29.9 ± 5.2	31.0 ± 5.2	<0.001
**BMI class**					<0.001
<25	157 (100)	1337 (84.1)	261 (10.8)	7 (1.4)	
*25.0–29.9*	0 (0)	134 (8.4)	1117 (46.2)	259 (50.3)	
≥*30.0*	0 (0)	119 (7.5)	1038 (43.0)	249 (48.3)	
**Physical activity**					<0.001
*Sedentary*	0 (0)	6 (0.4)	127 (5.3)	292 (56.7)	
*Moderate*	48 (30.6)	516 (32.4)	1023 (42.3)	104 (20.2)	
*High*	109 (69.4)	1068 (67.2)	1266 (52.4)	119 (23.1)	
DASH score	32.9 ± 1.7	27.9 ± 3.5	26.6 ± 3.1	25.8 ± 3.4	<0.001
**DASH categories**					<0.001
*Low*	0 (0)	151 (9.5)	337 (14.0)	109 (21.2)	
*Medium*	0 (0)	1173 (73.8)	2047 (84.7)	406 (78.8)	
*High*	157 (100)	266 (16.7)	32 (1.3)	0 (0)	
**Alcohol consumption**					<0.001
*Never/light*	45 (28.7)	349 (22.0)	611 (25.3)	97 (18.8)	
*Moderate*	112 (71.3)	1234 (77.6)	1639 (67.8)	159 (30.9)	
*Heavy*	0 (0)	7 (0.4)	166 (6.9)	259 (50.3)	
**Smoking status**					0.009
*Non-smoker*	99 (63.1)	901 (56.7)	1500 (62.1)	275 (53.4)	
*Current smoker*	15 (9.5)	197 (12.4)	224 (9.3)	66 (12.8)	
*Ex-smoker*	43 (27.4)	492 (30.9)	692 (28.6)	174 (33.8)	
History of CV events	4 (2.6)	108 (6.8)	157 (6.5)	33 (6.4)	0.145
Diabetes	6 (3.8)	68 (4.3)	327 (13.5)	92 (17.9)	<0.001
Dyslipidemia	72 (45.9)	705 (44.3)	1371 (56.8)	311 (60.4)	<0.001
Chronic kidney disease	5 (3.2)	23 (1.5)	28 (1.2)	7 (1.4)	0.229
**Anti-hypertensive medications**					<0.001
*Mono-therapy*	107 (68.6)	1052 (67.7)	1383 (58.3)	287 (56.4)	
*Dual therapy*	46 (29.5)	410 (28.4)	785 (33.1)	167 (32.8)	
*Triple therapy or more*	3 (1.9)	92 (5.9)	204 (8.6)	55 (10.8)	

Data are mean ± SD for quantitative variables or percent for categorical.

*P* from logistic regression model adjusted for age.

Abbreviations: BMI, body mass index (Kg/m^2^); BP, blood pressure; CV, cardiovascular; DASH, dietary approach to stop hypertension; HRT, hormone replacement therapy; SD, standard deviation.

### Uncontrolled BP and unhealthy behaviors

The association between uncontrolled hypertension and dietary adherence, physical activity, body mass index (BMI), alcohol consumption and the number of unhealthy behavior is described in Table 4 for men and in a supplementary table for women. In men, there was no major difference between the associations found after adjustment for age, monthly income and education (model 1), and after additional adjustment for diabetes and dyslipidemia (model 2). In other words, associations found to be significant in model 1 remained significant in model 2. However, in women, the association between individual unhealthy lifestyle factors and uncontrolled hypertension did not achieve statistical significance and is available in supplementary table 1.

**Table 4:** Association between uncontrolled hypertension and the number of unhealthy behaviors in men.

Term	Model 1	P value	Model 2	P value
**DASH**		**0.019**		**0.017**
*High*	1.00 (ref)	—	1.00 (ref)	—
*Medium*	1.26 [1.04–1.52]	0.020	1.26 [1.04–1.53]	0.018
*Low*	1.41 [1.11–1.79]	0.005	1.41 [1.11–1.79]	0.004
*Low/medium vs. high*	1.13 [0.97–1.34]	0.113	1.14 [0.97–1.35]	0.105
**Physical activity**		**0.041**		**0.031**
*High*	1.00 (ref)	—	1.00 (ref)	—
*Moderate*	0.86 [0.77–0.97]	0.012	0.86 [0.77–0.96]	0.009
*Sedentary*	0.91 [0.76–1.08]	0.285	0.90 [0.76–1.08]	0.274
*Moderate/sedentary vs. high*	0.87 [0.78–0.98]	0.013	0.87 [0.78–0.97]	0.010
**BMI**		**<0.001**		**<0.001**
*<25*	1.00 (ref)	—	1.00 (ref)	—
*25.0–29.9*	1.23 [1.07–1.40]	0.002	1.25 [1.09–1.43]	0.001
*≥30.0*	1.54 [1.33–1.79]	<0.001	1.57 [1.35–1.83]	<0.001
*≥25 vs.<25*	1.33 [1.18–1.51]	<0.001	1.35 [1.19–1.53]	<0.001
**Alcohol consumption**		**0.003**		**0.003**
*Never/light*	1.00 (ref)	—	1.00 (ref)	—
*Moderate*	1.07 [0.91–1.27]	0.410	1.08 [0.91–1.27]	0.367
*Heavy*	1.33 [1.09–1.61]	0.004	1.34 [1.10–1.63]	0.003
*Heavy vs. moderate/never*	1.25 [1.09–1.44]	0.001	1.25 [1.09–1.44]	0.001
**Nb. of unhealthy behaviors**		**<0.001**		**<0.001**
*0*	1.00 (ref)	—	1.00 (ref)	—
*1*	1.12 [0.74–1.71]	0.585	1.11 [0.73–1.69]	0.612
*2*	1.39 [0.92–2.09]	0.120	1.38 [0.91–2.08]	0.123
*3 or more*	1.66 [1.08–2.52]	0.019	1.67 [1.09–2.53]	0.018

Abbreviations: BMI, body mass index (Kg/m^2^); DASH, dietary approach to stop hypertension.

*Model 1:* logistic regression model adjusted for age, education level, monthly income.

*Model 2:* logistic regression model adjusted for age, education level, monthly income, diabetes, and dyslipidemia.

Regarding dietary adherence, men reporting low or medium dietary adherence had a 1.26-fold (adjusted odds ration (ORa) 1.26, 95% confidence interval (CI) 1.04–1.53) and 1.41-fold (ORa 1.41, 95% CI 1.11–1.79) increase of the odds of uncontrolled hypertension compared to those with high dietary adherence. Similarly, the odds of uncontrolled hypertension increased by 1.25-fold (1.09–1.43) and 1.57-fold (1.35–1.83) in overweight and obese men, respectively, compared to a normal BMI. In addition, there was a significant association between alcohol consumption and uncontrolled hypertension (p = 0.003); men consuming alcohol heavily had an increase of the odds of uncontrolled hypertension compared to light/never drinkers by 1.34-fold (ORa 1.34, 95% CI 1.10–1.63). The association remained significant when dichotomizing the variable and comparing heavy drinking to moderate/light drinking (ORa 1.35, 95% CI 1.09–1.44; p = 0.01). In addition there was a significantly increasing age-adjusted mean systolic BP across light, moderate and heavy drinking in both sexes: in women the mean systolic BP ± SD across categories was 134.6 ± 4.8, 135.5 ± 4.2 and 136.1 ± 4.2, respectively (p < 0.001) and in men it was 134.9 ± 4.6, 135.7 ± 4.0 and 136.4 ± 3.6, respectively (p < 0.001). As for physical activity, there was an unexpected inverse relationship between sedentary level physical activity and uncontrolled hypertension. Moreover, the prevalence of uncontrolled hypertension increased with the number of unhealthy lifestyle factors in men only (p < 0.001). Those reporting three or more unhealthy behaviors had 1.67-fold (1.09–2.53) increase of the odds of uncontrolled hypertension.

## Discussion

Findings of our study show, from a population-based perspective, that modifiable unhealthy lifestyle factors such as heavy alcohol consumption, overweight and non-adherence to dietary recommendations were associated with increased risk of uncontrolled hypertension in hypertensive treated individuals. The association was significant in men only, but even after adjustment for socio-demographic characteristics and cardiovascular risk factors. Also, a dose-effect relationship was noticeable by increased odds of uncontrolled hypertension with higher number of unhealthy behaviors; compared to none, three or four unhealthy lifestyle factors had 1.7-fold increased odds of uncontrolled hypertension in men. To our knowledge, these results are among the few to estimate the quantitative extent to which individual and combined unhealthy lifestyle factors influence the risk of uncontrolled hypertension in pharmacologically treated patients.

Compared to a normal BMI, we found overweight and obesity to be strongly associated with uncontrolled hypertension in men, increasing its odds by 1.25-fold and 1.57-fold respectively. This association has been described elsewhere; one study in South Korea conducted on individuals being treated for hypertension and taking regularly their antihypertensive medications, found that overweight patients were less likely to have their BP under control compared with those whose body weight was normal (ORa 0.44; p < 0.05)^9^. Similarly, the Framingham Heart Study reported that among treated subjects, increasing age, obesity and the presence of left ventricular hypertrophy were associated with lack of systolic BP control. The authors suggested that public health efforts should be directed at achieving goal BP levels especially in patients who are older, are overweight or have target organ damage^16^.

There was no significant association between physical activity and uncontrolled hypertension in women. While in men, surprisingly, the multivariable analysis models found a weak but significant inverse association between physical activity and uncontrolled hypertension, whereby moderate and moderate-to-sedentary physical activity level compared to high level (as reference) were negatively associated with uncontrolled hypertension. In general, this is not in accordance with results of observational studies that reported a strong relationship between physical activity and BP control. Ham and Young, found that low physical activity (compared to high level) to be associated with poor BP control among hypertensive treated individuals^9^. Other studies argued that moderate intensity aerobic exercise lowers BP in patients with hypertension and reduces the need for antihypertensive medication^17,18^. Although a dose-dependent relationship was not seen in our study, yet we found a protective relationship between moderate level physical activity and uncontrolled hypertension. Several aspects of the physical activity score we used could explain our divergent results. In fact we calculated a score that is not commonly used or is reproducible in the literature, which could have led to an inadequate estimation of physical activity level. Moreover, the score does not consider the metabolic equivalent (MET) of the reported physical activity, resulting in a distinct classification. Further studies are necessary to assess this aspect.

Concerning adherence to dietary recommendations, low or medium adherence to the dietary approaches to stop hypertension (DASH)-diet was found to increase the odds of uncontrolled hypertension in men only. Few studies evaluated the association between a dietary approach and BP control in uncontrolled hypertensive individuals. One randomized controlled trial conducted on hypertensive patients with type 2 diabetes and uncontrolled hypertension, demonstrated that a DASH-diet combined with increased daily walking promotes a clinically relevant reduction in ambulatory BP monitoring^19^. On the other hand, most research studied the BP lowering effect of a DASH-diet in pre-hypertensive and hypertensive patients. For example, the DASH collaborative research group found that adopting a DASH-diet in patients with hypertension substantially lowers systolic and diastolic BP by 11.4 and 5.5 mmHg, respectively^20^, suggesting that such BP reductions can help in achieving adequate BP control. Our study demonstrated that non-adherence to dietary recommendation is associated with uncontrolled hypertension, while measuring the extent of the effect of the association. Accordingly, our findings suggest that lifestyle modifications involving the adoption of a DASH-style diet offer an important approach in the treatment of hypertension.

We identified a strong association between heavy alcohol consumption and uncontrolled hypertension. Men who drank alcohol heavily had 1.34-fold increase in the odds of poor BP control. This association has been reported in previous studies. Ham *et al*. reported that heavy alcohol consumption defined as consumption of more than 60 g for men and 40 g for women during a single drinking session, was independently associated with poor BP control at the 140/90 threshold in a sample of hypertensive treated South Koreans^9^. In addition, a number of studies described an apparent and direct association between heavy alcohol intake and elevated BP^21,22^ that can result in exceeding recommended BP goals. One Japanese study found that in heavy drinkers, systolic and diastolic BP was 2.3/2.0 mmHg higher in heavy drinkers than in non-drinkers^21^. Differences between gender in drinking behavior, pattern and beverage choice could have influenced the lack of association in women. Nevertheless, an association between heavy drinking and high BP was once again demonstrated, supporting limitation of alcohol consumption recommendation, in the hypertensive treated population.

We found a dose-effect relationship between the number of unhealthy behaviors and uncontrolled hypertension; the likelihood of uncontrolled hypertension increased nearly linearly with 1, 2, 3 or more, unhealthy lifestyle factors, but reached statistical significance with 3 or more factors. Few epidemiological studies evaluated the role of modifiable lifestyle factors on BP control among treated individuals, or assessed their combined effect. In fact, collective unhealthy behaviors may have synergistic effect on BP control, emphasizing the importance of studying their combined effect. In patients with uncontrolled hypertension and type 2 diabetes, the combination of increasing physical activity and following a DASH-diet had a major reduction in systolic BP values of approximately 15 mmHg, as compared with a reduction of 3 mmHg in the control group^19^. Importantly, with such BP reductions, more than half of the patients in the intervention group reached the recommended goals for daytime ambulatory BP monitoring^19^. BP reductions with a combination of lifestyle factors were discussed in previous studies on hypertensive patients, but there was no reference to the use of anti-hypertensive medications. For example, a systematic review of randomized controlled trials on patients with elevated blood pressure, reported BP reductions of 5.5 mmHg after a combination of interventions including physical activity, diet and weight loss, compared to 5.0 mmHg for improved diet, 4.6 mmHg for exercise and 3.5 mmHg for alcohol restriction^23^. In a recent separate analysis, we evaluated the extent to which unhealthy behavior influence the development of hypertension in the general population, and found that in both sexes, unhealthy behavior (as described in this study) significantly increased the odds of hypertension. We also reported that a combination of two and three-or-more unhealthy behaviors resulted in an increased odds of hypertension by 1.77-fold and 2.29-fold respectively in men, and by 1.71-fold and 2.14-fold respectively in women^24^. Our current study show that a combination of unhealthy behavior is independently associated with uncontrolled hypertension in hypertensive treated individuals. These findings along with the previous ones^24^ further support the important influence of lifestyle factors on high BP, in different populations and regardless of the presence of other risk factors. However, when comparing both studies, the magnitude of the effect of this association appears stronger on the development of hypertension than on BP control, suggesting that other factors should also be considered when evaluating determinants of BP control in individuals pharmacologically treated for hypertension.

Our study also pointed out gender differences as to these associations. Few observational studies evaluated the determinants of uncontrolled hypertension using a gender stratified analysis^9,25^ and they were not particularly on unhealthy behaviors. Discrepancy between sexes could be explained by differences in lifestyle habits between men and women as well as to the influence of other confounding factors^6–8^ such as other socioeconomic factors (employment, marital status), other diseases (chronic kidney disease) other behavioral factors (such as salt intake and stress) and adherence to anti-hypertensive medications. In addition, some data suggest that sex-related characteristics such as the level of sex hormones may influence the results^26^. Although further research is needed to clarify this difference, nevertheless adopting a global healthy lifestyle is important for prevention of cardiovascular diseases and should be encouraged in the general population^14^.

Lastly, age-and-gender-adjusted results found current smoking to be associated with decreased prevalence of uncontrolled hypertension. Epidemiological studies describe discrepancy with regards to effect of smoking on uncontrolled BP; some studies reported smoking to negatively influence BP control^27,28^, while others found no association^9,25,29^ and showed that office BP is not lowered by smoking cessation^30^. Further research can help yield more conclusive results. Nevertheless, smoking is an unhealthy behavior and a major risk of CVD and cancer; smoking cessation recommendations should be provided to all hypertensive individuals for the prevention of CVD including stroke, myocardial infarction and peripheral artery disease^1,2,14^.

The main strength of our study is using data from CONSTANCES, which was designed adopting a population-based method including a large randomly selected sample of participants that ensures sufficient power. Moreover, BP measurements were collected following standardized protocols and data were gathered using different methods such as validated questionnaires and national databases, resulting in lack of missing information. Additionally, we explored the combined effect of unhealthy behaviors and performed an analysis stratified by sex. However, some limitations should be addressed. Reverse-causality bias is inherent in cross-sectional analyses, preventing the confirmation of a causal relationship between unhealthy behaviors and uncontrolled hypertension. In addition, our study might be subject to selection bias because of the selection effect related to voluntary participation, also because agricultural and self-employed workers were not included in CONSTANCES. Misclassification bias is also possible, since lifestyle behaviors were self-reported. Moreover, the time frame between recent medication adjustment and BP measurement was not taken into consideration. In fact, antihypertensive medication is a confounding factor in BP measurements, with epidemiologic data on BP often compromised by the effects of antihypertensive medications^31^, and certainly recent changes. Nevertheless, this is common in epidemiologic studies of cross-sectional design; prospective data from CONSTANCES can help in considering this point. Lastly, excessive salt intake is considered an unhealthy behavior, but we weren’t able to study its effect on uncontrolled hypertension because quantitative data on salt intake are not available since dietary habits were evaluated using a non-quantitative food frequency questionnaire.

In conclusion, findings of this study provide supportive evidence of the individual and combined effect of unhealthy behaviors on uncontrolled hypertension. Unhealthy lifestyle described as heavy alcohol drinking, non-adherence to dietary recommendations and overweight increased the likelihood of uncontrolled hypertension, which was further increased with a higher number of unhealthy factors. Our findings revealed that the associations were significant in men only, suggesting the presence of other factors influencing uncontrolled hypertension. Although further research is needed to clarify the reasons behind the gender-based differences, our findings contribute to epidemiologic data of utmost importance in the management of hypertension, especially in the presence of limited data on the effect of lifestyle factors on hypertension control. From a population-based perspective, our study advocates that public health strategies should promote improvement of modifiable behaviors through a multidisciplinary lifestyle changes approach, which could offer considerable benefits in the treatment and control of hypertension, particularly in men.

## Methods

### Study design and study population

Details concerning objectives and study design of the cohort CONSTANCES (http://www.constances.fr/index_EN.php) have been previously published^15,32^. Briefly, CONSTANCES is a prospective epidemiological cohort composed of randomly selected adult participants aged 18–69 years at inception affiliated with the French National Health Insurance Fund database (CNAM; General scheme which covers 85% of the general French population) following a sampling scheme stratified on age, gender, socioeconomic status and region of France.

Volunteers who agreed to participate in the study had to fill self-administered questionnaires and were invited to attend to one of the 22 selected health-screening centers (HSCs) to benefit from a comprehensive health examination. They were also linked through French health administrative and national social databases. Through these different sources, social, demographic, health, behavioral, occupational, biological, and anthropometric data were collected. All the participants included in the CONSTANCES cohort have signed an informed consent form. This research follows the tenets of the Declaration of Helsinki and was approved by the National Data Protection Authority (*Commission Nationale Informatique et Libertés*; CNIL) and the Institutional Review Board of the National Institute for Medical Research and the local Committee for Persons Protection (*Comité de Protection des Personnes*).

### Study participants

The present study is a cross-sectional analysis on participants who were known to have hypertension recorded by the physician or measured during the medical examination at the HSC and receiving antihypertensive medications. From a total of 87,808 volunteer participants recruited between February 2012 and January 2018 and to whom data was available through linkage with the health insurance administrative database, 10,764 subjects met the above definition and were eligible to be included in the current analysis. We excluded 54 participants with BMI <18kg/m^2^ and we therefore analyzed 10,710 hypertensive treated participants.

### Uncontrolled blood pressure

BP measurements were done based on standardized operational procedures (SOPs)^33^ during the clinical examination at the HSC. Systolic BP and diastolic BP were measured using an automated oscillometric sphygmomanometer, in each arm at 2 minutes interval and after 5 minutes of rest. The arm giving the highest systolic BP was considered the reference arm and a third BP measure was taken after 1 minute of rest, the average of these 2 measurements was considered. Uncontrolled BP was defined as mean systolic BP ≥140 mm Hg and/or mean diastolic BP ≥90 mm Hg^34^.

### Behavioral risk factors definitions

Lifestyle factors were assessed through validated self-administered questionnaires. They were described and classified based on a previous study evaluating the association between unhealthy behavior and risk of hypertension^24^. Similarly, unhealthy behavior was defined like in the original study^24^. The lifestyle factors are presented also here.

*Alcohol consumption* was determined considering the quantity and type of alcoholic beverages consumed the previous week^35^. We subsequently defined alcohol consumption as never/light (0–3 glass/week (0–30 g/week) for men and 0–2 (0–20 g/week) for women), moderate (4–21 (40–210 g/week) glass/week for men and 3–14 (30–140 g/week) for women) and heavy drinkers (>21 glass/week (>210 g/week) for men and >14 (>140 g/week) for women)^36^. Heavy drinking was considered an unhealthy behavior.

*Physical activity* was assessed through three questions that considered the frequency of transferring, leisure time activity and sports^37^. We assigned 0, 1, or 2 points for each question based on an escalating frequency of activity, then a score of 0–6 was calculated and physical activity level was classified as sedentary (0–2), moderately active (3–4) and highly active (5–6). Sedentary level was considered an unhealthy behavior^37^.

*Dietary assessment* was done through a validated 52-items food frequency questionnaire (FFQ) from which a DASH score was constructed based on 8 food groups or nutrients for which consumption should be increased (fruits, vegetables, nuts and legumes, low-fat dairy, whole grains) or reduced (sodium, sweetened beverages, red and processed meats)^38^. Consumption of each dietary component was divided into quintiles, and participants’ intakes were assigned 1–5 points according to a gender-specific intake ranking^38,39^. Component scores were summed, and an overall DASH score ranging from 8–40 was calculated. The DASH score was subsequently collapsed to tertiles for analysis; a higher tertile indicating a higher dietary quality, adherence to dietary recommendations was subsequently categorized into low, medium and high. We considered low/medium dietary adherence an unhealthy behavior.

*Body mass index* (BMI, kg/m^2^) was calculated at the HSC, then categorized into three classes: normal (≤25 kg/m^2^), overweight (25 kg/m^2^<BMI <30 kg/m^2^), and obese (≥30 kg/m^2^). We considered overweight/obese (BMI >25 kg/m^2^) an unhealthy behavior.

Accordingly, participants could exhibit 0 (none), 1, 2, 3, or 4 unhealthy behaviors.

### Covariates

Covariates were defined and classified as the original analysis^24^ and they included the following. Education level was collected according to the International Standard Classification of Education (ISCED)^40^ and was then classified into three levels: High school diploma or less (≤13 years of education), undergraduate degree (14–16 years of education) and postgraduate degree (≥17 years of education). Marital status was categorized into couple life or single (including widowed or separated/divorced). Household monthly income was categorized into:<1000; 1000–2099; 2100–4199; ≥ 4200 euros per month.

Blood glucose, triglycerides and total cholesterol were measured by taking fasting blood samples at the HSC. Diabetes mellitus status was based on either receiving anti-diabetic medication or a fasting blood glucose concentration greater than or equal to 7 mmol/L. Dyslipidemia was defined as having a fasting plasma total-cholesterol or triglycerides level of ≥6.61 mmol/L (255 mg/dL) or >1.7 mmol/L (150 mg/dL) respectively. History of CV diseases was considered as any self-reported previous diagnosis of angina pectoris, myocardial infarction, cerebrovascular accident or peripheral artery disease^14^. Chronic kidney disease was defined as known proteinuria or decreased renal function (creatinine clearance <60 ml/min calculated by the Cockroft-Gault equation) for more than 3 months^41^, or a chronic kidney disease diagnosed by biopsy or renal ultrasound and confirmed by a nephrologist.

### Statistical analysis

Descriptive analysis was performed using counts and percentages or mean ± SD. Each characteristic was compared between subjects with controlled and uncontrolled hypertension using logistic regressions adjusted for age and sex. In addition, we compared characteristics of subjects according to the number of unhealthy behaviors using logistic regressions adjusted for age and stratified by sex. Also with logistic models, we estimated the association between uncontrolled hypertension and unhealthy behaviors. In a first step, models were adjusted for age, education and monthly income (model 1). In a second step, models were further adjusted for diabetes and dyslipidemia (model 2). Initially, we performed separate models for each unhealthy behavior using categorical variables and binary variables. Then, we examined the association between uncontrolled hypertension and the number of unhealthy behaviors (0- ≥ 3) independently associated with control of hypertension. General Linear Model was used to study age-adjusted mean systolic BP across ascending number of unhealthy factors. Adjusted odds ratios (ORa) were presented along with 95% confidence interval (CI), all statistical analyses were performed with SAS 9.4 (SAS Institute) and p ≤ 0.05 was considered significant.

## Supplementary information

Supplementary information.

## Data Availability

The datasets generated during and/or analyzed during the current study are available from the CONSTANCES principal investigator (marie.zins@inserm.fr) provided that the procedures described in the CONSTANCES Charter (http://www.constances.fr/charter) are fulfilled.
