# Supplementary material for: Unhealthy behaviors and risk of uncontrolled hypertension among treated individuals-The CONSTANCES population-based study
Source: Sci Rep. 2020 Feb 5;10:1925. doi: 10.1038/s41598-020-58685-1 (PMC7002708; doi:10.1038/s41598-020-58685-1)
Supplement: Supplementary file 1 — Supplementary information. [file 41598_2020_58685_MOESM1_ESM.docx]

**Unhealthy behaviors and risk of uncontrolled hypertension among treated individuals-The CONSTANCES population-based study**

**Short title:** Unhealthy Behavior and Uncontrolled Hypertension

**Authors:**

### Michelle Cherfan^1,2*^, Alexandre Vallée^3,4*^, Sofiane Kab^5^, Pascale Salameh^6,7^, Marcel Goldberg^3,5^, Marie Zins^3,5^, Jacques Blacher^1,3,4^

^*^authors who contributed equally to the work

**Affiliations:**

^1^Nutritional Epidemiology Research Unit (EREN), Inserm U1153, Inra U1125, Cnam, Crnh, Paris 13 University Sorbonne Paris Cite, Bobigny, France

^2^Faculty of Pharmacy, Lebanese International University, Beirut, Lebanon

^3^Faculty of Medicine, Paris-Descartes University, Paris, France

^4^Diagnosis and Therapeutic Center, Hypertension and Cardiovascular Prevention Unit, Hôtel-Dieu Hospital; AP-HP, Paris, France

^5^Population-based Epidemiological Cohorts Unit, Inserm, UMS011, Villejuif, France

^6^Faculty of Public Health, Lebanese University, Fanar, Lebanon

^7^Institut National de Santé Publique, Epidémiologie Clinique et Toxicologie (INSPECT-LB), Beirut, Lebanon.

**Corresponding author:**

Jacques Blacher, MD, PhD

Faculty of Medicine, Paris-Descartes University, AP-HP; Hôtel-Dieu University Hospital

Diagnosis and Therapeutic Center, Hypertension and Cardiovascular Prevention Unit

Address: Place du Parvis Notre-Dame, 75004 Paris, France

Tel: +01 42 34 82 34; Fax: 01 42 34 86 32

Email: [jacques.blacher@aphp.fr](mailto:jacques.blacher@aphp.fr)

**Sources of Funding**

The Constances Cohort Study was supported and funded by the Caisse nationale d’assurance maladie (CNAM). The Constances Cohort Study is an “Infrastructure nationale en Biologie et Santé” and benefits from a grant from ANR (ANR-11-INBS-0002) and from the Ministry of Research. Constances is also partly funded by MSD, AstraZeneca and Lundbeck.

**SUPPLEMENTARY TABLE**

**Table 1. Association between uncontrolled hypertension and the number of unhealthy behaviors in women**

| ***Term*** | ***Model 1*** | ***P value*** | ***Model 2*** | ***P value*** |
| --- | --- | --- | --- | --- |
| ***DASH*** |  | **0.222** |  | **0.223** |
| High | 1.00 (ref) | - | 1.00 (ref) | - |
| Medium | 1.17 [0.91-1.50] | 0.233 | 1.15 [0.89-1.49] | 0.277 |
| Low | 1.19 [0.98-1.46] | 0.083 | 1.19 [0.98-1.46] | 0.085 |
| Low/medium vs. high | 1.01 [0.84-1.20] | 0.972 | 1.01 [0.85-1.21] | 0.863 |
| ***Physical activity*** |  | **0.118** |  | **0.147** |
| High | 1.00 (ref) | - | 1.00 (ref) | - |
| Moderate | 1.14 [1.01-1.29] | 0.046 | 1.13 [0.99-1.28] | 0.063 |
| Sedentary | 0.99 [0.80-1.23] | 0.972 | 0.99 [0.79-1.22] | 0.893 |
| Moderate/sedentary vs. high | 1.10 [0.98-1.25] | 0.092 | 1.09 [0.97-1.24] | 0.124 |
| ***BMI*** |  | **0.701** |  | **0.608** |
| <25 | 1.00 (ref) | - | 1.00 (ref) | - |
| 25.0-29.9 | 1.06 [0.92-1.22] | 0.436 | 1.04 [0.90-1.19] | 0.614 |
| ≥30.0 | 1.01 [0.87-1.16] | 0.938 | 0.96 [0.82-1.12] | 0.600 |
| ≥25 vs. <25 | 1.03 [0.91-1.17] | 0.603 | 1.01 [0.88-1.14] | 0.968 |
| ***Alcohol consumption*** |  | **0.502** |  | **0.512** |
| Never/light | 1.00 (ref) | - | 1.00 (ref) | - |
| Moderate | 1.02 [0.89-1.18] | 0.755 | 1.03 [0.89-1.19] | 0.664 |
| Heavy | 1.14 [0.91-1.44] | 0.252 | 1.15 [0.91-1.44] | 0.250 |
| Heavy vs. moderate/never | 1.12 [0.91-1.38] | 0.257 | 1.12 [0.92-1.37] | 0.284 |
| ***Nb. of unhealthy behaviors*** |  | **0.267** |  | **0.314** |
| 0 | 1.00 (ref) | - | 1.00 (ref) | - |
| 1 | 1.34 [0.96-1.89] | 0.090 | 1.34 [0.96-1.89] | 0.088 |
| 2 | 1.33 [0.95-1.86] | 0.094 | 1.30 [0.93-1.83] | 0.124 |
| 3 or more | 1.45 [1.01-2.11] | 0.047 | 1.40 [0.97-2.05] | 0.070 |

**Abbreviations:** BMI, body mass index (Kg/m^2^); DASH, dietary approach to stop hypertension

**Model 1:** logistic regression model adjusted for age, education level, monthly income.

**Model 2:** logistic regression model adjusted for age, education level, monthly income, diabetes, and dyslipidemia.
